# Neoadjuvant Chemotherapy with Concurrent Letrozole for Estrogen Receptor-Positive and HER2-Negative Breast Cancer: An Open-Label, Single-Center, Nonrandomized Phase II Study (NeoCHAI)

**DOI:** 10.3390/cancers16183122

**Published:** 2024-09-10

**Authors:** Heejung Chae, Sung Hoon Sim, Youngmi Kwon, Eun-Gyeong Lee, Jai Hong Han, So-Youn Jung, Seeyoun Lee, Han-Sung Kang, Yeon-Joo Kim, Tae Hyun Kim, Keun Seok Lee

**Affiliations:** 1 Department of Internal Medicine, Center for Breast Cancer, Hospital, National Cancer Center, 323 Ilsanro, Goyang 10408, Republic of Korea; 2 Cancer Data Center, National Cancer Control Institute, National Cancer Center, 323 Ilsanro, Goyang 10408, Republic of Korea; 3 Division of Rare and Refractory Cancer, Research Institute, National Cancer Center, 323 Ilsanro, Goyang 10408, Republic of Korea; 4 Department of Pathology, National Cancer Center, Hospital, 323 Ilsanro, Goyang 10408, Republic of Korea; 5 Department of Surgery, Center for Breast Cancer, Hospital, National Cancer Center, 323 Ilsanro, Goyang 10408, Republic of Korea; 6 Division of Clinical Research, Research Institute, National Cancer Center, 323 Ilsanro, Goyang 10408, Republic of Korea; 7 Center for Proton Therapy, National Cancer Center, 323 Ilsanro, Goyang 10408, Republic of Korea

**Keywords:** hormone-receptor positive breast cancer, early breast cancer, neoadjuvant treatment, endocrine therapy, chemotherapy

## Abstract

**Simple Summary:**

Neoadjuvant chemotherapy is the standard of care for locally advanced breast cancer; however, it is less effective in hormone receptor (HR)-positive tumors. Therefore, we aimed to evaluate the safety and efficacy of adding an aromatase inhibitor to standard neoadjuvant chemotherapy in patients with stage II or stage III HR-positive, HER2-negative breast cancer. Adding letrozole to standard neoadjuvant chemotherapy did not significantly increase the pathologic complete response (pCR) rate; however, it enhanced the overall response rate with acceptable safety. Further research is necessary to determine the optimal neoadjuvant therapy for HR-positive, HER2-negative breast cancer to address the ongoing unmet need compared with recent advances in clinical outcomes observed in HER2-positive and triple-negative subtypes.

**Abstract:**

The role of combining neoadjuvant endocrine therapy with conventional chemotherapy remains unclear; therefore, we conducted an open-label, single-center, nonrandomized phase II trial to assess the effect of this combination. Patients with previously untreated stage II or III HR-positive, HER2-negative breast cancer received concurrent letrozole 2.5 mg with standard neoadjuvant chemotherapy. The primary endpoint was pathologic complete response (pCR) at the time of surgery. We used Simon’s minimax two-stage design; a pCR rate > 6% was necessary at the first stage to continue. Between November 2017 and November 2020, 53 women were enrolled in the first stage of the trial. Their median age was 49 years (range, 33–63), and 60% of them were premenopausal. Subsequently, 66% and 34% of patients with clinical stages II and III, respectively, were included; 93% had clinically node-positive disease. Two patients (4%) achieved pCR after neoadjuvant chemo–endocrine treatment, which did not satisfy the criteria for continuing to the second stage. The overall response rate was 83%. During the median follow-up of 53.7 months, the 3-year disease-free survival and overall survival rates were 87% and 98%, respectively. Neutropenia was the most common grade 3/4 adverse event (40%), but rarely led to febrile neutropenic episodes (4%). Myalgia (32%), nausea (19%), constipation (17%), heartburn (11%), oral mucositis (9%), and sensory neuropathy (9%) were frequently observed, but classified as grade 1 or 2. No deaths occurred during preoperative treatment. The addition of letrozole to standard neoadjuvant chemotherapy was safe and beneficial in terms of overall response rate, but did not provide a higher pCR rate in locally advanced HR-positive, HER2-negative breast cancer. Further research is needed to enhance neoadjuvant treatment strategies for this cancer subtype.

## 1. Introduction

Breast cancer is the most frequent cancer in women worldwide and a leading cause of cancer-related deaths, with 2,309,000 new cases and 666,000 deaths reported in 2022 [1]. The hormone receptor (HR)-positive, human epithelial receptor 2 (HER2)-negative subtype accounts for approximately 70% of all breast cancer cases and is mostly diagnosed in early, resectable stages [2]. Neoadjuvant chemotherapy with anthracycline, taxane, and alkylator-based regimens is the current standard of care for locally advanced breast cancer. However, it tends to be less effective in patients with HR-positive tumors [3,4]. The rate of HR-positive tumors achieving a pathologic complete response (pCR) to chemotherapy is significantly lower (7–16%) than those of HER2-positive or triple-negative breast cancer (30–50%).

Since the advent of tamoxifen, a selective estrogen receptor modulator, in the 1970s, endocrine therapy agents with different mechanisms, including aromatase inhibitors and selective estrogen receptor degraders, have been developed and have become the mainstay treatment options for HR-positive breast cancer in both adjuvant and metastatic settings [5]. Although several studies have investigated the efficacy of neoadjuvant endocrine treatment, its clinical application is generally limited to patients unfit for chemotherapy [6]. The SWOG 8814 trial reported that the concurrent administration of tamoxifen with chemotherapy in the adjuvant setting was associated with marginally inferior disease-free survival and overall survival compared with sequential tamoxifen after chemotherapy [7]. Similarly, other studies reported that concurrent administration of tamoxifen and chemotherapy generally increased response rates, but did not significantly improve survival outcomes [8,9,10,11,12]. Additionally, beyond the limited additive efficacy, the combination of chemotherapy with tamoxifen is typically contraindicated owing to the increased risk of serious thromboembolic complications [13]. Therefore, chemotherapy alone has been the standard of care for the neoadjuvant systemic treatment of HR-positive breast cancer.

However, these earlier studies were mainly conducted with tamoxifen and chemotherapy regimens that did not include anthracyclines or taxanes. Non-steroidal aromatase inhibitors (NSAIs) have now become the current standard of care in postmenopausal and high-risk premenopausal women, showing a lower risk of recurrence compared with tamoxifen [14,15,16]. Findings from previous trials using tamoxifen should not be extrapolated to NSAIs in the absence of sufficient research. Additionally, the chemotherapy regimens used in past trials differ from contemporary standard regimens that include anthracycline, cyclophosphamide, and docetaxel [17]. In contrast, the combination of adjuvant capecitabine and endocrine therapy was permitted in the CREATE-X trial without robust scientific evidence [18]. Therefore, evaluating the impact of the concurrent use of aromatase inhibitors and anthracycline plus taxane-based chemotherapy is important. Consequently, this open-label, single-center, non-randomized trial aimed to assess the efficacy and safety of letrozole combined with neoadjuvant anthracycline plus cyclophosphamide followed by docetaxel in patients with high-risk HR-positive early breast cancer.

## 2. Materials and Methods

### 2.1. Study Design and Eligibility

In this single-center, single-arm, non-randomized, open-label prospective phase II trial, patients newly diagnosed with clinical stage IIA–IIIC (any cT2-3N0M0 or cT1-3N+M0), HR-positive, HER2-negative breast cancer who were candidates for neoadjuvant treatment were enrolled. Additional eligibility criteria included age between 19 and 70 years, no prior history of chemotherapy, an Eastern Cooperative Oncology Group performance status of ≤2, and adequate organ function. The key exclusion criteria were inflammatory breast cancer, history of other malignant cancer, cerebrovascular disease, cardiovascular disease, peripheral arterial disease or thromboembolism, the presence of low cardiac function (ejection fraction <55%), and a diagnosis of any intestinal malabsorption disease. Prior systemic treatment, radiotherapy, or therapeutic surgery was not permitted before enrollment. This study was approved by the Institutional Review Board of the National Cancer Center in Korea (NCC2017-0110), and all participating patients provided written informed consent. This study was registered on clinicaltrials.gov on 13 April 2018 (NCT03497702).

### 2.2. Treatment and Assessment

**Figure 1:**
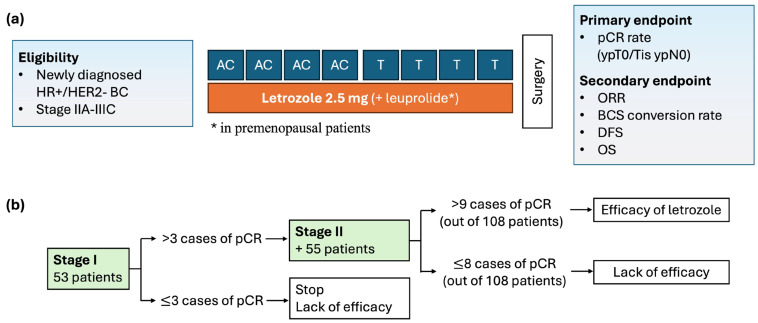
Study design. Treatment schema (**a**) and Simon’s minimax two-stage design (**b**).

Eligible patients received eight cycles of standard neoadjuvant chemotherapy (four cycles of doxorubicin 60 mg/m^2^ plus cyclophosphamide 600 mg/m^2^ every 3 weeks, followed by four cycles of docetaxel 75 mg/m^2^ every 3 weeks) with concurrent letrozole 2.5 mg. Ovarian function suppression was preceded by leuprolide (3.75 mg) every 4 weeks in premenopausal women (Figure 1). Treatment was discontinued if disease progression or unacceptable toxicity occurred. Patients underwent definitive surgery within 5 weeks after the last cycle of neoadjuvant chemotherapy. Adjuvant treatments were administered at the physician’s discretion according to local guidelines.

Before the start of neoadjuvant treatment, surgeons assessed each patient’s resection extent plan based on the baseline physical examination and imaging studies. Treatment response was evaluated after the 4th and 8th cycles of neoadjuvant treatment using a breast imaging study. Subsequently, the surgical plan was re-evaluated after the completion of neoadjuvant treatment and compared with the initial plan. pCR was assessed according to definitions of the pathological stages determined by a pathologist specializing in breast cancer. Follow-up visits and imaging studies were conducted until 5 years after surgery to monitor for recurrence and death. Toxicity profiles during the neoadjuvant phase, including laboratory values and symptoms, were evaluated at every visit and graded by Common Terminology Criteria for Adverse Events version 4.0.

### 2.3. Endpoints

The primary endpoint of the trial was pCR, defined as a pathological stage ypT0/is, ypN0 at the time of definitive surgery. Secondary endpoints included response rate, downstaging to breast-conserving surgery, disease-free survival (DFS), overall survival (OS), and safety. Tumor response was determined according to the revised Response Evaluation Criteria in Solid Tumors version 1.1. DFS was defined as the time from definitive surgery to disease progression that was local (both invasive and in situ) or distant recurrence, secondary cancer, or death from any cause, whichever occurred first. OS was defined as the time between definitive surgery and death from any cause. When recurrence or death was not observed, survival time was censored at the date of the last follow-up visit.

### 2.4. Statistical Analysis

Simon’s two-stage design was employed to determine the sample size. The pCR rate following neoadjuvant chemotherapy in HR-positive, HER2-negative breast cancer was 6.6% (p0 = 0.06) in our retrospective cohort. Combining letrozole with standard neoadjuvant chemotherapy was anticipated to increase the pCR rate to 12% (p1 = 0.12). With a power of 0.80 (beta = 0.20) and a one-sided alpha level of 0.10, we estimated that 53 patients were required for stage I. More than three pCR cases were necessary to progress to stage II, in which an additional 55 patients would be recruited. The benefit of letrozole would be considered significant if more than nine pCR cases out of 103 patients were observed. Considering a 5% dropout rate, we planned to enroll 114 participants.

Survival outcomes were estimated using the Kaplan–Meier method. All statistical analyses were conducted using the Statistical Package for the Social Sciences version 14.0 (SPSS Inc., Chicago, IL, USA). The survival graph in the figure was created using GraphPad Prism 10 (GraphPad Software, Boston, MA, USA).

## 3. Results

### 3.1. Patients and Treatment

Between November 2017 and November 2020, 53 women were enrolled in the first stage of the trial. Their baseline characteristics are summarized in Table 1. Their median age was 49 years (range, 33–63), and premenopausal women comprised 60% of participants. Patients with clinical stages II (n = 35, 66%) and III (n = 18, 34%) were included, with most (n = 49, 93%) of them presenting clinically node-positive disease. Invasive ductal carcinoma was observed in most patients (n = 48, 91%). More than two-thirds (n = 37, 70%) of these patients exhibited tumors with a high ki-67 (≥ 14%) at baseline biopsy. Eleven patients with a high risk of genetic predisposition (e.g., early onset breast cancer or a strong family history of cancer) underwent germline genetic testing as per guidelines; however, none were found to carry germline mutations, including BRCA1/2.

Most (n = 51, 96%) patients completed eight cycles of chemotherapy concurrently with letrozole, except for two patients who proceeded to surgery without completing four cycles of docetaxel owing to disease progression (Table 2). Chemotherapy delays were observed in 5 patients (9%), whereas 12 (23%) patients required dose reduction. Adherence to letrozole was well established, with an administration rate exceeding 90% in 93% (n = 49) of patients.

### 3.2. Efficacy

Two patients (4%) achieved pCR after treatment with a combination of letrozole and neoadjuvant chemotherapy, which did not satisfy the criteria for continuing to the second stage. Based on imaging studies, the response rate was 83% with a disease control rate of 96% (Table 2). Out of fourteen patients who were initially considered mastectomy candidates, four patients (29%) were able to undergo breast-conserving surgery following downsizing from neoadjuvant treatment. Two patients (4%) could not complete eight cycles of neoadjuvant treatment owing to disease progression and proceeded with surgery sooner than planned. The pCR rate and response rate were not significantly associated with menopausal status, Ki-67 index, clinical stage, T stage, or LN involvement in the subgroup analysis.

**Figure 2:**
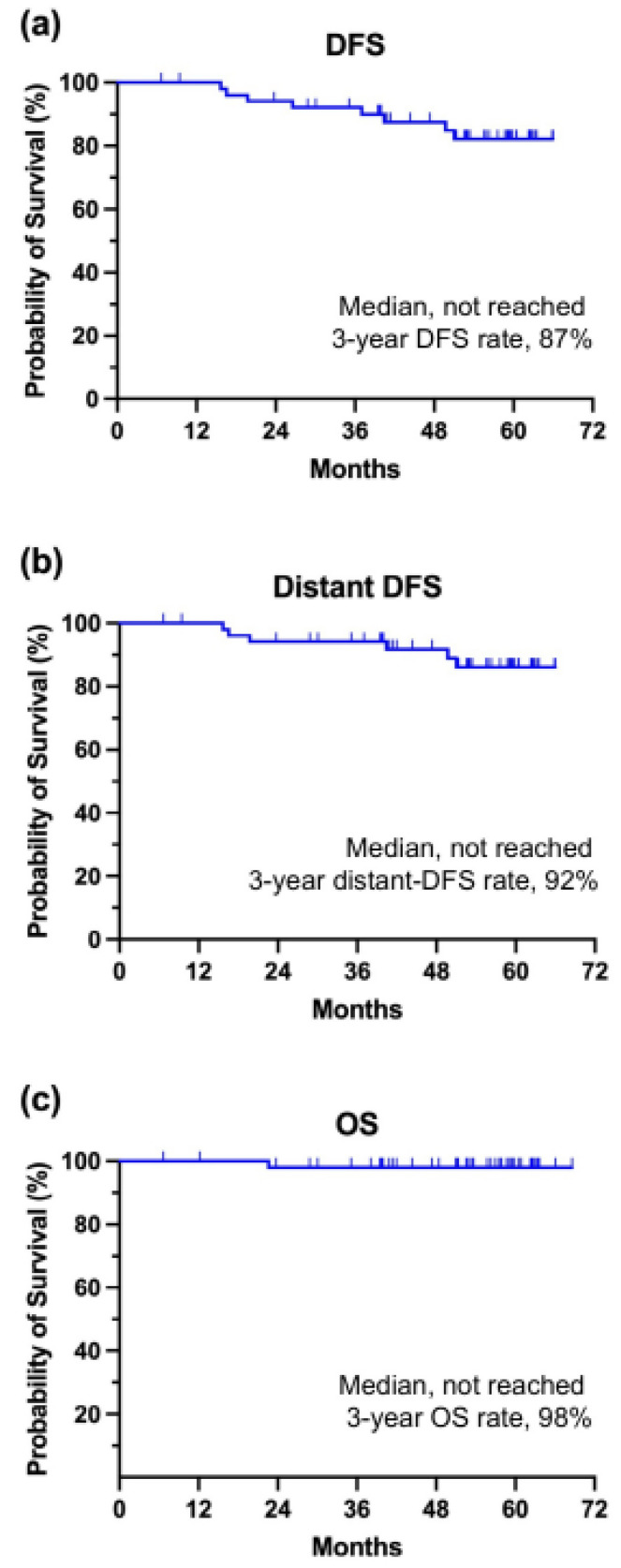
Survival outcomes for all patients. Disease-free survival (DFS) (**a**), distant disease-free survival (**b**), and overall survival (OS) (**c**).

During the median follow-up of 53.7 months, the 3-year DFS rate was estimated to be 87%, with a 3-year distant DFS rate of 92% (Figure 2). Six patients (11%) experienced distant metastasis of breast cancer, whereas two patients developed stage I lung cancer and contralateral ductal carcinoma in situ. Notably, recurrence was not associated with the response to previous neoadjuvant chemo–endocrine therapy, as all recurrent patients showed a partial response to neoadjuvant letrozole plus chemotherapy. The 3-year estimated OS rate for the entire population was 98%, with only one death attributed to brain metastasis of breast cancer.

### 3.3. Safety

Neutropenia was the most common grade 3/4 adverse event (40%), but it rarely led to febrile neutropenic episodes (4%) (Table 3). Myalgia (32%), nausea (19%), constipation (17%), heartburn (11%), oral mucositis (9%), and sensory neuropathy (9%) were frequently observed, but all were classified as grade 1 or 2. No patients experienced fracture events or thromboembolisms throughout the entire follow-up period. No deaths occurred during preoperative treatment.

## 4. Discussion

According to current international guidelines, neoadjuvant chemotherapy based on anthracycline, docetaxel, and alkylator regimens is recommended in patients with high-risk HR-positive, HER2-negative early breast cancer. In contrast, neoadjuvant endocrine treatment can increase locoregional treatment options in highly selected patients with luminal-A-like tumors [19,20]. However, the combination of chemotherapy and endocrine therapy has not yet been established. Our results demonstrate that endocrine therapy with letrozole, in addition to standard neoadjuvant chemotherapy, is well tolerated, but does not provide a higher pathologic response rate in early HR-positive, HER2-negative breast cancer. In the 53 patients included in this open-label, non-randomized trial, the pCR rate was 4%, with 3-year DFS and OS rates of 87% and 98%, respectively.

Previous studies investigating the efficacy of combining letrozole and chemotherapy as neoadjuvant therapy reported inconsistent results [21,22]. In a phase III trial involving 101 postmenopausal women in Iran, the addition of letrozole to neoadjuvant chemotherapy resulted in a higher pCR rate of 26.5% compared with the control arm (10.2%, *p* = 0.049), contradictory to our findings. However, the Iranian study needs careful interpretation, in that a third of the participants had non-HR-positive breast cancer, and all patients received a non-taxane-containing chemotherapy regimen (5-FU, doxorubicin, and cyclophosphamide), which is not considered an optimal regimen in current practice. In contrast, our results are consistent with those of a Japanese phase II study wherein 28 patients were randomized to two different treatment groups: concurrent anthracycline-taxane-based chemotherapy combined with endocrine therapy using an aromatase inhibitor (plus leuprolide in premenopausal patients) and chemotherapy alone. The results showed no statistically significant difference in the pCR rate between the groups (12.5% vs. 8.3%, *p* = 1.0). Long-term follow-up for survival outcomes is necessary, as pCR is not a well-established surrogate endpoint for DFS and OS in HR-positive, HER2-negative breast cancer [23].

We demonstrated a significantly higher response rate (83%) with concurrent letrozole and chemotherapy in HR-positive, HER2-negative breast cancer compared with previous studies on chemotherapy alone, which showed a clinical response rate of 60–70% [24,25]. Unlike the impact of combining letrozole with chemotherapy on pCR and long-term survival outcomes, which is inconclusive, the improvement in response rate from this combination has been consistently observed across several studies [21,22,26]. Tumor response can be beneficial for patients who require downstaging to ensure negative margins for their bulky tumors and wish to preserve their breasts. Our study had a conversion rate of approximately 30% for initially being ineligible for breast-conserving surgery to becoming eligible due to neoadjuvant chemo–endocrine treatment. A smaller number of changes in surgical plans observed in our study compared to the previous literature may be attributed to the inherently high rates of breast-conserving surgery at the National Cancer Center Korea; our surgeons possess a high level of expertise in cancer surgery and tend to employ broader indications for breast-conserving surgery.

The safety profile and dose reduction/interruption incidence for the combination of letrozole with chemotherapy were similar to those previously reported for neoadjuvant chemotherapy [27,28]. In our study, grade 3/4 toxicity consisted mainly of hematologic adverse events (neutropenia rate, 40%), but this rarely became clinically critical (febrile neutropenia, 4%). Notably, no thromboembolic event was observed in patients during the treatment phase. Previous studies have reported a significantly higher risk of thromboembolism associated with the combination of tamoxifen and chemotherapy compared with tamoxifen monotherapy (13.6% vs. 2.6%), which has posed a major challenge to the concurrent use of tamoxifen and chemotherapy [10]. Aromatase inhibitors, which are known to be less thrombogenic than tamoxifen, are also safer when used in combination with chemotherapy [29]. There was no treatment-related mortality, and most adverse events were well tolerated with appropriate dose modification and active supportive care.

CDK4/6 inhibitors, which inhibit the progression of the cell cycle from the G1 to the S phase, are considered the most promising combination partner to endocrine treatment. The three CDK4/6 inhibitors—palbociclib, ribociclib, and abemaciclib—have been approved for use in combination with aromatase inhibitors and fulvestrant as first line treatments of HR-positive, HER2-negative metastatic breast cancer, showing greater efficacy than endocrine therapy alone or cytotoxic chemotherapy [30,31,32,33,34,35]. Recently, the combination of CDK4/6 inhibitors and endocrine therapy has been the focus of several neoadjuvant trials [36,37,38,39]. These studies have shown that molecular downstaging, as indicated by factors like the Ki-67 proliferation index, preoperative endocrine prognostic index score, and PAM50 low-risk-of-relapse, can be effectively achieved through this combination strategy. However, improvement in clinical response rates or pCR rates have not been significant. This underscores the continuing unmet need in neoadjuvant treatment for HR-positive, HER2-negative breast cancer, especially when compared with the significantly improved outcomes seen in HER2-positive or triple-negative breast cancer in recent years.

Nonetheless, the limitations of this study include its non-randomized, single-center design and the relatively small sample size. We utilized historical controls to formulate hypotheses and compare our results to draw more definitive conclusions about the optimal use of endocrine treatment and its best combination partner in the early breast cancer setting. Additionally, given the biology of the hormone receptor-positive subtype, which carries an extended risk of recurrence over a long period, further follow-up for our study population is warranted [40]. Our study included 60% premenopausal patients, as breast cancer in Korea is most commonly diagnosed in the 40–49 year age group, significantly younger than the peak age observed in Western countries (>65 years) [41]. This is a key strength of our study, as our results could represent breast cancers in younger patients, which are known to be more aggressive and characterized by distinct molecular profiles, including lower proportions of ER+ and luminal A subtypes, but higher proportions of luminal B and lower ER gene expression [42].

## 5. Conclusions

In this study, neoadjuvant concurrent chemo–endocrine therapy resulted in a low pCR rate, not reaching the prespecified pCR efficacy target. Consequently, the addition of letrozole to standard anthracycline and taxane-based neoadjuvant chemotherapy should not be recommended for patients with locally advanced breast cancer. Nonetheless, our findings suggest that combining an aromatase inhibitor with neoadjuvant chemotherapy could enhance the response rate with an acceptable safety profile. Further investigation is necessary to determine the optimal neoadjuvant systemic therapy strategy for patients with HR-positive, HER2-negative breast cancer.

## Figures and Tables

**Table 1:** Clinical characteristics of patients.

Characteristic	Total (n = 53)
Age (years), median (range)	49 (33–63)
Sex, Female	53 (100%)
Menopausal status	
Premenopausal	32 (60%)
Postmenopausal	21 (40%)
Histologic subtype	
Invasive ductal carcinoma	48 (91%)
Invasive lobular carcinoma	5 (9%)
Ki-67	
< 14%	16 (30%)
≥ 14%	37 (70%)
Stage	
II	35 (66%)
III	18 (34%)
Node	
Negative	4 (8%)
Positive	49 (93%)
Initial surgery plan	
Breast-conserving surgery candidate	39 (74%)
Mastectomy candidate	14 (26%)

**Table 2:** Treatment summary and clinical outcomes for all patients.

	Total (n = 53)
Chemotherapy	
Complete without interruption	37 (70%)
Dose reduction or delay	14 (26%)
Cessation	2 (4%)
Letrozole compliance	
≥ 90%	49 (93%)
< 90%, ≥ 80%	2 (4%)
< 80%	2 (4%)
Overall response rate	
Partial response	44 (83%)
Stable disease	7 (13%)
Progressive disease	2 (4%)
Surgery	
Breast-conserving surgery	42 (79%)
Mastectomy *	11 (21%)
Pathologic complete response	2 (4%)
Recurrence	
Distant	6 (11%)
Local	0

* One patient was eligible for breast-conserving surgery but opted for mastectomy due to concerns regarding recurrence and a desire to avoid radiotherapy.

**Table 3:** Adverse events during the treatment period.

	Total (n = 53)
Neutropenia, grade 3/4	21 (40%)
Febrile neutropenia	2 (4%)
Myalgia	17 (32%)
Nausea	10 (19%)
Constipation	9 (17%)
Heartburn	6 (11%)
Oral mucositis	5 (9%)
Sensory neuropathy	5 (9%)
Death	0

## Data Availability

Datasets generated or analyzed during the current study are not publicly available due to the risk of re-identification when providing detailed information about each patient’s clinical information. Still, they are available from the corresponding author upon reasonable request.
